# Lengthening of the humerus with intramedullary lengthening nails—preliminary report

**DOI:** 10.1007/s11751-017-0286-6

**Published:** 2017-04-24

**Authors:** Julian Fürmetz, Søren Kold, Nikola Schuster, Florian Wolf, Peter H. Thaller

**Affiliations:** 1 3D-Surgery, Department of General, Trauma and Reconstructive Surgery, Munich University Hospital LMU, Germany, Nußbaumstraße 20, 80336 Munich, Germany; 2 Department of Orthopaedics, Aarhus University Hospital, Århus, Denmark

**Keywords:** Humerus lengthening, Intramedullary lengthening, Distraction osteogenesis, Lengthening nail, FITBONE, PRECICE

## Abstract

Distraction osteogenesis of the humerus with fully implantable lengthening is now possible since the diameter of the available nails was reduced to 10 mm and below. We report on the first intramedullary lengthening cases of the humerus with two different lengthening devices (FITBONE and PRECICE). Two different approaches and implantation techniques were used. We retrospectively reviewed clinical and radiographic data and pointed out results, pitfalls and complications of the procedure. Four adult patients with relevant length discrepancy of the humerus were treated with fully implantable systems in two centers between 2012 and 2015. Three patients were treated with FITBONE by an antegrade approach; one patient had lengthening with a PRECICE and a retrograde approach. Average nail lengthening was 55 mm (40–65 mm), and the average duration of lengthening was 70 days (52–95 days). The average distraction index was 0.72 mm/day (range 0.4–1.0 mm/day) or 12.5 days/cm (range 8.0–16.2 days/cm). The average consolidation index was 33.6 days/cm (range 25–45 days/cm). There was an implant failure (arrest) with the PRECICE. After consolidation and exchange with a technically improved implant, the course of treatment was uneventful. In patients with antegrade lengthening shoulder abduction decreased, and in the patient with the retrograde approach it improved but elbow extension decreased marginally. Reduced motion of the adjacent joints can be a major problem in intramedullary lengthening of the humerus. This first case series in the field of a rare indication suggests that lengthening of the humerus by fully implantable lengthening nails might be a valuable alternative to lengthening with external fixation. Main advantage of the PRECICE technology is the possible shortening in-between of lengthening.

## Introduction

Today, distraction osteogenesis has become a crucial tool in limb lengthening and deformity correction. Regarding leg lengthening, it has already been described at the beginning of the last century and since the work of Ilizarov it has been more and more understood and brought into clinical use [1].

Compared to leg length discrepancies, arm length discrepancies are less frequent and subsequent secondary damage is lower [2]. But, functional impairment, cosmetic reasons and muscular problems may be an indication for a correction. Lengthening of the humerus was first reported by Dick and Tietjen in 1978 using a Wagner fixator, plating and bone grafting [3]. Since then, different ways of external fixation have been described for lengthening of the humerus [4–9]. Currently, circular frames like the Ilizarov frame and monolateral fixators are the most common fixation techniques and lead to similar results [8, 9]. The results indicate a significant improvement of function, and therefore, lengthening of the humerus is not just a cosmetic procedure.

Regarding the lower limb, lengthening by intramedullary devices gained popularity over the past decades. In the first decades of intramedullary lengthening, the diameter of the available lengthening devices was too large for smaller bones and thus for the upper limb. To this date, there is only one other report in the literature about lengthening of the humerus by an intramedullary device, but lacking to provide any results or details [10].

Lengthening nails have many advantages such as no pin site infections, lesser soft-tissue damage and pain, better joint movement and more patient comfort compared to external devices [10–16]. In the past years, technological progress made smaller diameters, as low as 8.5 mm possible [10].

Presently, the most frequently implanted systems are the FITBONE nail, (WITTENSTEIN Intens GmbH, Igersheim, Germany) and the PRECICE nail (Ellipse Technologies Inc., CA, USA) after the ISKD was withdrawn from the market 2009 and the last PHENIX nail was implanted in 2013 [17]. The diameters range from 11 to 13 mm (FITBONE) and 8.5 to 12.5 mm (PRECICE). The FITBONE nail is a motorized (electromotive) system which was developed in 1990 [11]. The PRECICE nail is a magnetically actuated, mechanical system and was introduced to the market in 2011 [10].

In the following, we present two different approaches and implantation techniques regarding intramedullary lengthening of the humerus with two different systems (FITBONE and PRECICE).

## Patients and methods

### Study patients

We reviewed four patients who underwent five intramedullary lengthening procedures between 2012 and 2015. Three patients underwent three lengthenings with FITBONE, and one patient underwent two lengthenings with PRECICE. All patients were fully informed about the nature of the procedure and the technology involved. All patients explicitly wanted an internal lengthening procedure and declined an external lengthening procedure. Details regarding patient age, sex, etiology, length discrepancy, surgery, lengthening details and complications were tabulated (see Table 1). Calibrated humerus AP radiographs were performed to obtain the humerus length discrepancy before the procedure.

**Table 1:** Patients and methods

Patient	M.J.	T.K.	Y.G.	G.K.
Sex	Male	Female	Male	Male
Age	19	19	27	51
History	Erb–Duchenne-type obstetric palsy	Erb–Duchenne-type obstetric palsy	Traumatic growth arrest in childhood	Posttraumatic shortening after complex fracture and nonunion
Disorders	Neck pain, back pain, functional deficit	Neck pain, functional deficit	Functional deficit	Posttraumatic stress disorder, functional deficit
Side	Left	Right	Left	Left
Preoperative humerus shortening (mm)	50	65	65	40
Type of nail	TAA 1160 Tibia FITBONE	TAA 1160 Custom straight FITBONE	TAA 1160 Custom straight FITBONE	PRECICE retrograde femur, 2nd generation P2 and P2.1
	Diameter 11 mm Length 225 mm Stroke 60 mm	Diameter 11 mm Length 205 mm Stroke 60 mm	Diameter 11 mm Length 205 mm Stroke 60 mm	Diameter 8.5 mm Length 215 Stroke 50 mm
Osteotomy height from tip of greater tuberculum (mm)	120	130	90	200; 190
Distraction index (mm/day)	0.8	0.6	0.8	0.45 and 1.0
Consolidation index (days/cm)	27.5	25	45	40.0 and 30.3
Problems/obstacles	Proximal humeral head migration, stop of lengthening and remaining shortage of 10 mm	Z-plastic of biceps tendon due to flexion contracture of the elbow	Removal of receiver and chord penetrating rotator cuff to gain better ROM	Early consolidation after “crown breakage” (PRECICE P2)

Two patients suffered from a posttraumatic shortening of the humerus, one in childhood (patient Y.G.; FITBONE) and one adult patient (G.K. PRECICE). The other two patients (FITBONE) had unilateral humeral shortening caused by Erb–Duchenne-type obstetric palsy. All FITBONE patients had a minimum follow-up of 6 months after nail removal. The PRECICE patient (G.K.) has still the nail in situ, and follow-up is 18 months after nail implantation. The case of M.J. has been published previously [18].

### Treatment strategy

The first three patients received lengthening with the FITBONE nail using an antegrade approach. The PRECICE lengthening nail (second generation) was used twice for lengthening of the humerus of patient G.K. using a retrograde approach. The second PRECICE nail was the technical improved version P2.1 which has no ‘thru-slots’ or tack welds at the end of the proximal nail [10]. To protect the radial nerve from damage during osteotomy, a careful soft-tissue dissection is performed to the bone, and a drill guide and soft-tissue protectors are placed to perform safe drilling and chiseling.

### Antegrade approach

**Fig. 1:**
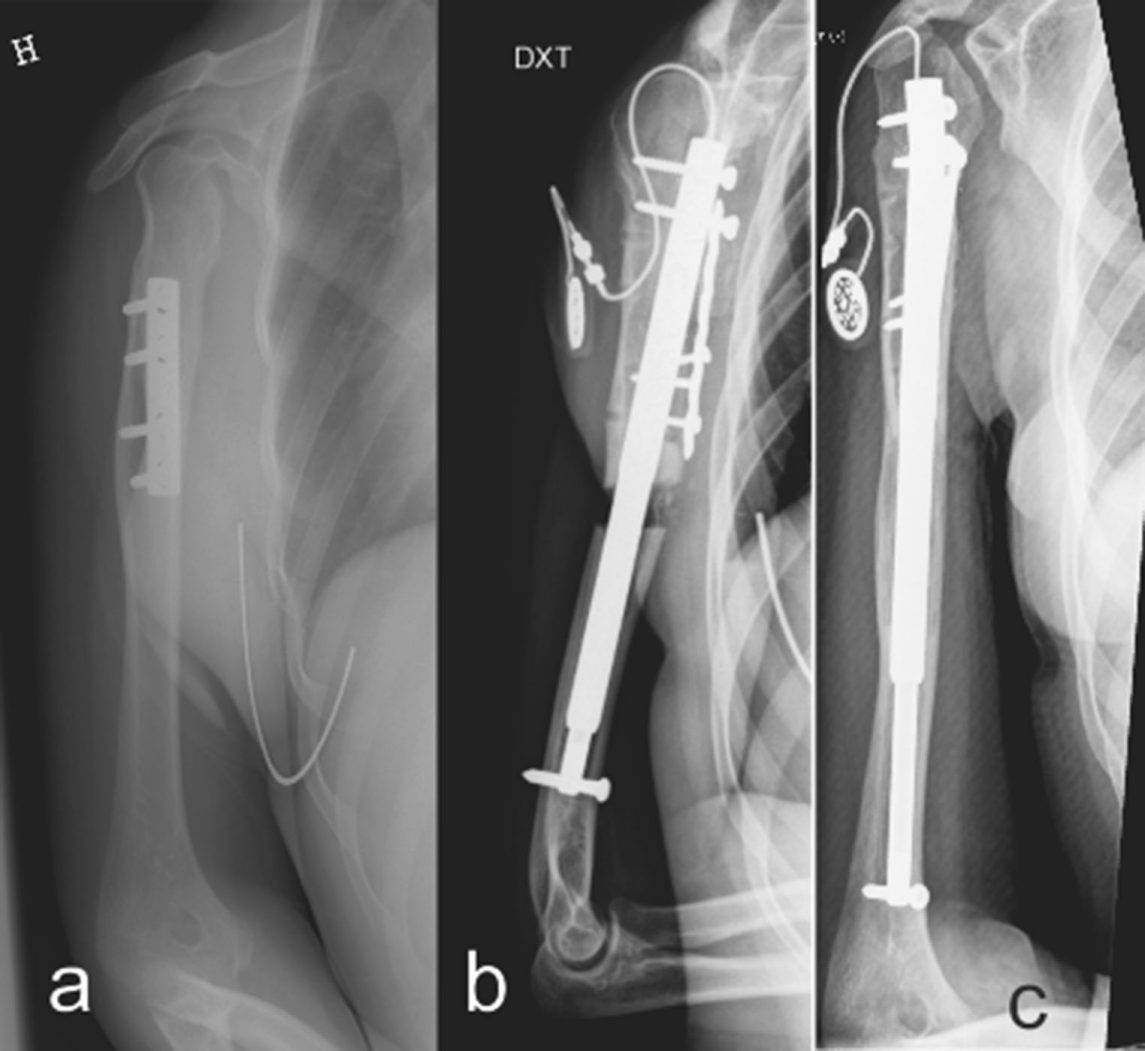
**a** Preoperative radiograph of 19-year-old patient T.K. with 65 mm shortening of the right humerus after Erb–Duchenne-type obstetric palsy. The patient had at the age of 12 years an external rotation osteotomy fixed with a plate. **b** Follow-up radiograph after lengthening has been initiated by the inserted FITBONE. After the previously inserted plate was removed at the time of FITBONE insertion a cortical defect existed. In order to securely lock the proximal part of the nail, a new plate was fixed in good cortical bone distally and one of the proximal locking screws were inserted through a plate hole. **c** Radiograph after consolidation of 6-cm lengthened humerus

For antegrade humeral nailing, an anterolateral transdeltoid approach provided access to the humeral canal, and the supraspinatus tendon was split in the direction of its fibers. A protective steel sleeve was passed through the split supraspinatus tendon prior to reaming. Reaming was performed with straight reamers. In two patients (M.J. and Y.G.) the osteotomy was completed with a chisel after careful dissection and predrilling through a small lateral incision. In patient T.K. a previously inserted humeral plate had to be removed prior to nail insertion (see Fig. 1), and the drill and chisel osteotomy was completed through this larger approach. The motor unit inside the nail was connected to a subcutaneously placed receiver by a cord passing through the split in the supraspinatus tendon.

During distraction procedure, the patient had to hold a transmitter over the receiver to activate the motor unit inside the lengthening nail. Lengthening was started at the eighth postoperative day at a maximum rate of 1/3 mm three times per day.

### Retrograde approach

**Fig. 2:**
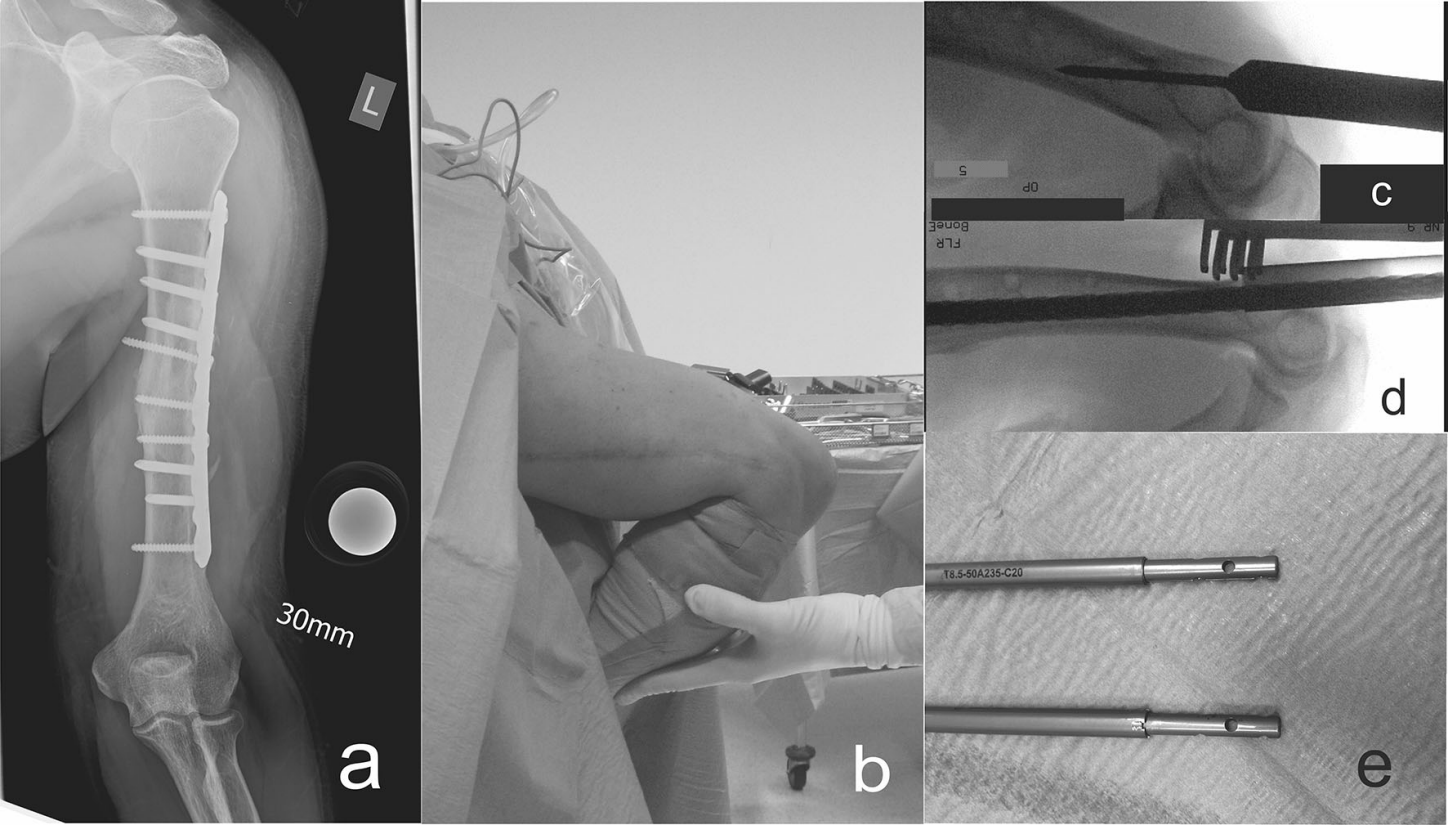
**a** Preoperative radiograph of patient G.K. with 40 mm shortening of the left humerus after a successfully treated nonunion; **b** intraoperative supine positioning of the patient for retrograde approach; **c** and **d** steel sleeves and rigid reamers for preparing the intramedullary canal; **e** crown breakage of the first PRECICE (P 2) below, predistracted nail above (P 2.1)

**Fig. 3:**
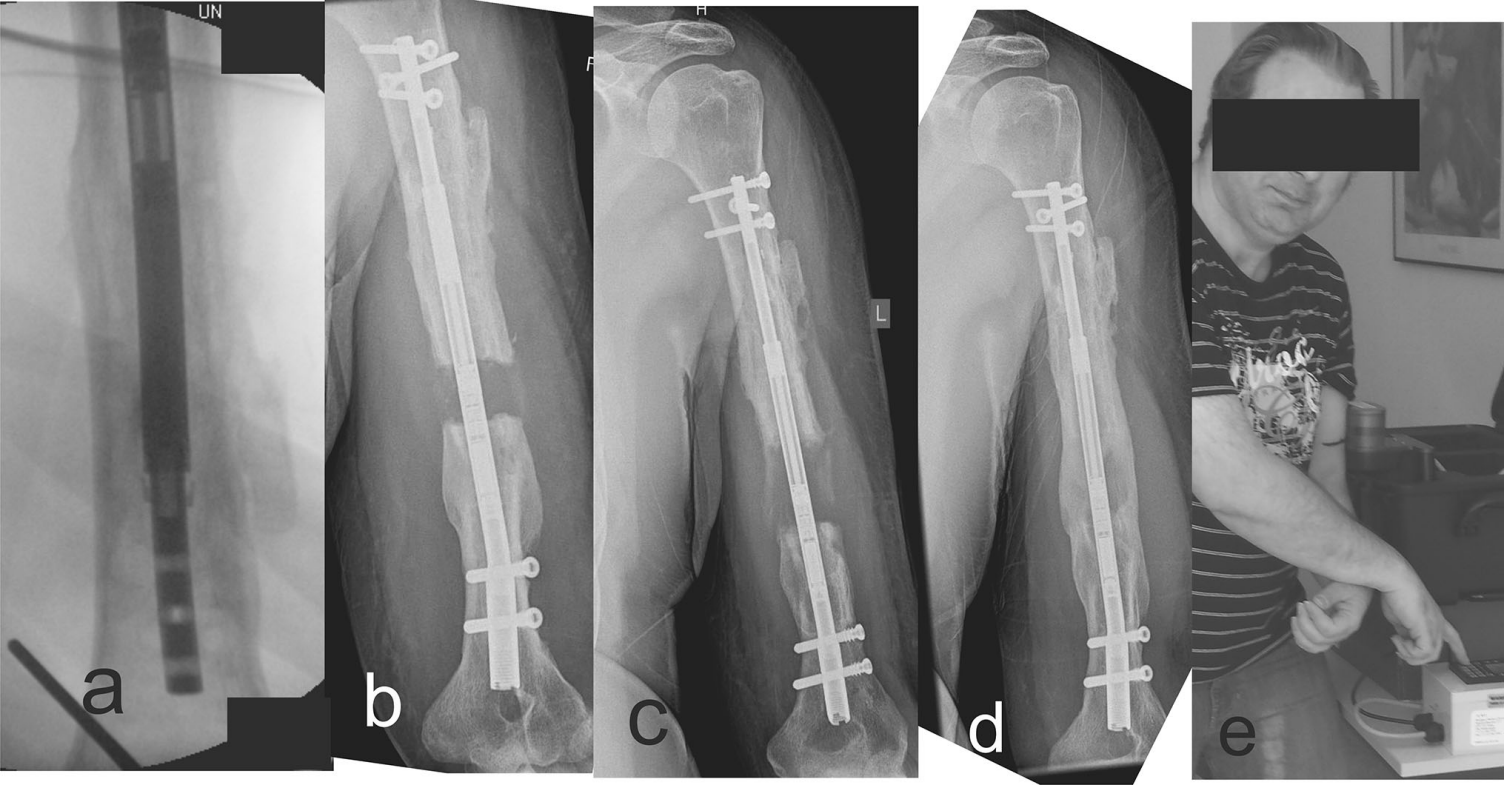
**a** Intraoperative radiograph of crown breakage of patient G.K.; **b**–**d** radiographs during the lengthening progress with the new PRECICE P2.1; **e** positioning of the external remote controller for lengthening

Patient G.K. had a posttraumatic decreased shoulder function. A retrograde approach was chosen to protect the rotator cuff and to avoid proximal migration of the humeral head (see Figs. 2, 3).

For retrograde technique in supine position, the supinated patient’s elbow has to be fully flexed (up to 140°). Through a small triceps split, the guide wire enters the cortex of the olecranon fossa roof. A steel sleeve was passed through the triceps tendon, and the reaming was performed with straight reamers. Two 3-mm K-wires were placed in the proximal and distal part of the humerus for torsion control. Through a minimally invasive lateral approach below, the insertion of the deltoid muscle a drill bit osteotomy was performed and completed with a small chisel. Lengthening with the PRECICE nail was started at the fifth postoperative day at rate of 1/3 mm, three times per day. After detailed instruction we provided the patient an external magnet controller for his personal use. For the lengthening procedure, the controller was placed on a table hooked into its transport box in an upright position (see Fig. 3).

### Postoperative care and follow-up

Before starting the lengthening procedure, a two-plane radiograph of the humerus and a calibrated AP radiograph from the osteotomy site were taken. During lengthening, the distraction was weekly controlled clinically and radiologically by calibrated radiographs of the osteotomy site and adjusted according to radiological signs of bone formation in the regenerate. To preserve the range of motion of the adjacent joints, physiotherapy was carried out on a regular basis. After the desired length was achieved, radiographs were taken every 2 weeks until full consolidation.

### Outcome

After finishing lengthening, the humeral length and the length of the regenerate were obtained on calibrated radiographs or CT Scouts. Consolidation was classified as three out of four cortices being present on the AP and lateral radiographs. Measurement of pre- and postoperative range of motion of the shoulder and elbow joints was performed by a senior surgeon. Subjective functional deficits in daily living, e.g., personal hygiene, clothing or type writing, were noted before and after the treatment. Additional procedures during the lengthening or consolidation period were noted. Complications which led to additional procedures during treatment were divided into implant- and non-implant-related complications.

### IRB approval

The study was carried out according to the Declaration of Helsinki. The Ethic Committee of the University of Munich approved this study with the ID number 8-16.

## Results

### Lengthening

Full lengthening was achieved in one patient (patient G.K.). Two patients (Y.G. and T.K.) had a residual discrepancy of 5 mm, and due to reduced shoulder motion (Y.G.) and elbow motion (T.K.) a minor difference was tolerated. In patient M.J. lengthening was terminated leaving the left humerus 1 cm short compared with the right side. After 4 cm of intramedullary nail lengthening (and a total of 9 cm lengthening due to prior 5 cm extramedullary lengthening with an Ilizarov fixator in childhood), the humeral head migrated proximal and the shoulder abduction declined, so lengthening was terminated.

Average nail lengthening was 55 mm (range 40–65 mm), and the average duration of lengthening was 70 days (range 52–95). The average distraction index was 0.72 mm/day (range 0.4–1.0 mm/day) or 12.5 days/cm (range 8.0–16.2 days/cm). Mean time to reach consolidation was 165 days after the distraction, and the average consolidation index was 33.6 days/cm (range 25–45 days/cm).

### Range of motion

Patient Y.G. showed no change in elbow motion. Shoulder abduction decreased 20°.

Patient M.J. showed no change in elbow motion. Shoulder abduction decreased 20° and flexion decreased 10°.

Patient T.K. improved elbow extension 10° after Z-plastic of the biceps tendon and botox injections two times. Shoulder motion did not change. There was an intended acute external rotation at the osteotomy of 10°.

Patient G.K. showed 5° decreased elbow extension, but shoulder abduction and flexion increased 10°.

### Complications

Irritation and pain caused by the cord, penetrating the rotator cuff, led to its removal in patient Y.G.

Lengthening in patient M.J. had to be stopped due to proximal migration of the humeral head and reduced shoulder function.

Reduction in elbow extension led to botox injections and a Z-plastic of the biceps tendon in patient T.K.

The first lengthening approach in patient G.K. failed after 10 mm of lengthening due to breakage of the crown (PRECICE P2) (see Fig. 2). After consolidation the implant was removed and a new osteotomy was performed 10 mm proximal to the regenerate. The nail was replaced by a 10 mm predistracted nail of the technically improved PRECICE P2.1 nail using the same locking options. At the beginning a transient radial nerve paralysis occurred, which recovered to the preoperative status. During the second lengthening approach, distraction progress declined in the radiographic controls compared to the controller. This can be related either to lack of transmission or to increase in resistance (early consolidation). We increased the distraction rate to 1.5 mm/day for 1 week. Finally, full lengthening was achieved, without facing other complications.

### Further outcome parameters

All patients achieved full consolidation. In the frontal and sagittal plane, no axis deviation was introduced by lengthening. We achieved one intended, but no unintended change in humeral torsion. Although no scoring system was used to precisely quantify the effect of treatment, all patients reported they were satisfied with the outcome. Patients reported about reduced neck pain and improved function in performing daily activities such as clothing and personal hygiene; resting arms at the table; improved function in type writing on the computer and improved function in steering the bicycle. The implant removal of two FITBONE nails (patients M.J. and T.K.) and of the first used PRECICE nail (patient G.K.) was carried out without any problems. Implant removal of one FITBONE nail (patient Y.G.) and the second PRECICE nail (patient G.K.) is planned for the near future.

## Discussion

Until now distraction osteogenesis for the humerus was only done by external fixation. Our report shows that lengthening of the humerus through intramedullary lengthening nails is possible. Evaluating the results we must consider the small sample size and our learning curve. Comparing with results of external lengthening, patients age must also be taken into account as most of the existing data are on lengthening the humerus in children or adolescents [4–6, 9].

Due to an implant failure of the PRECICE nail, one patient sustained early consolidation. Crown breakage of the PRECICE nail second generation P2 was reported in several oral presentations and once in literature [10]. The overall incidence of such crown breakage is not reported. The manufacturer solved this problem in a timely manner with a new design of the nail P2.1 which was released in December 2014.

The FITBONE nail showed one minor implant-related complication with following removal of the cable that connected the nail and subcutaneous receiver. Further possible risks of the FITBONE nail are breakage and running back of the telescopic part [16, 19]. The risk is higher in the lower limb due to weight bearing, but we recommend a close radiographic follow-up during lengthening and consolidation [16]. Close aftercare for adjusting the lengthening due to reduced distraction rate was important in the second lengthening approach (PRECICE P2.1) of patient G.K. As this problem was solved by increasing the daily distraction rate for 1 week, it seemed to be related to higher resistance likely caused by early consolidation. Not only early consolidation like in this case is a possible risk during lengthening, we also must be aware of regenerate insufficiency. Here the PRECICE nail brings one main advantage. The callus can be compressed easily by changing the lengthening direction without further operation and can then be lengthened again. This so-called accordion maneuver improves callus formation in distraction osteogenesis with external fixation and in the animal model [20]. We successfully applied it several times using the PRECICE in intramedullary lengthening of the lower limb.

Reduced range of motion in the adjacent joints was a major problem in our patients during the lengthening phase.

Two patients lost shoulder function. This may be due to the antegrade approach and a violation of the rotator cuff as it is described in fracture treatment [21, 22]. In fracture treatment loss of range of motion is described in antegrade nails for the shoulder and in retrograde nails for the elbow joint [23]. Both patients with a loss of shoulder abduction had change in shoulder anatomy due to Erb–Duchenne palsy. The preexisting shoulder instability might have allowed for the proximal migration of the humeral head resulting in loss of shoulder motion. By using a retrograde approach in lengthening, the violation of the rotator cuff can be prevented and the osteotomy can be performed distally to the insertion of the M. deltoideus, so that its function can be possibly maintained. It might be that the technical more challenging retrograde approach would have prevented loss of shoulder function. But if retrograde nailing is chosen great care must be taken to prevent secondary fractures, which is a frequently mentioned complication (2–10%) in retrograde fracture nailing [23].

Elbow motion can also be reduced by the entry point in retrograde nailing and due to increased biceps tension and less motion in the joint during the lengthening process. Additional procedures like botox injections or Z-plastic of the biceps tendon can help to restore or improve the elbow motion. Reduced range of motion in the adjacent joints can compromise the final lengthening result and points out the importance of intensive physiotherapy during the lengthening process.

Compared to reports regarding external lengthening of the humerus, we had a similar consolidation index (33 vs. 27–32 days/cm) [4–6, 8]. Reports with external Ilizarov ring fixation report about reduced shoulder or elbow motion in up to 7% of the cases [4]. Lengthening with monolateral fixation improved the function of the upper limb [8]. Fixation time varied between 7 and 9 months in case series with external fixation. Refracture rate after removal of the fixation was between 10 and 16% [4–6, 8]. Superficial pin track infections are common in external fixation with a risk up to 100%, but the risk of deep infection is low [24]. By using internal lengthening we need no external apparatus and avoid pin track infections, but the risk of deep infections in internal lengthening is not yet quantified. Additionally, the urge for removal of the internal implants is less than for external fixation which might lower the risk of refracture. However, the reduction in function in the adjacent joints was more pronounced with internal lengthening, and we had two implant-related interventions.

For both nails, breakage or malfunction has been reported [10, 19]. The FITBONE has a longer clinical history of more than 20 years. It is reported to unintentional backtrack and has the disadvantage of an additional cable [16, 19]. The PRECICE nail has thinner options (min. 8.5 mm) and the additional option for shortening (accordion-manoeuver) without further surgery [10, 25]. But no information exists about failure rates of the actual available PRECICE P2.1.

## Conclusions

Both implants (FITBONE and PRECICE) are possible options for intramedullary humerus lengthening and have different advantages and disadvantages. Reduced range of motion in the adjacent joints can be a problem during the lengthening. Both the entry point for the nail and the lengthening procedure can lead to reduced motion. As there are only few reports on humeral lengthening, we need more data for further evaluation. In the hands of an experienced surgeon, familiar with intramedullary lengthening devices the described techniques might be valuable treatment options in deformity correction of the upper limb.
